# Healthiness of Meat-Based Products in Comparison to Their Plant-Based Alternatives in the UK Market: A Packaging Evaluation

**DOI:** 10.3390/foods13213346

**Published:** 2024-10-22

**Authors:** Ruxandra Ciobotaru, Ayten Aylin Tas, Tabrez Ahmed Khan

**Affiliations:** 1 Department of Health Professions, Faculty of Health and Education, Manchester Metropolitan University, Manchester M15 6BG, UK; ruxandra_rxd@yahoo.com; 2 School of Agri-Food Technology and Manufacturing, College of Health Science, University of Lincoln, Lincoln LN6 7TS, UK

**Keywords:** plant-based foods, meat products, front of pack, back of pack, nutritional profiling, healthiness

## Abstract

This study evaluated the healthiness of meat products (n = 62) and their plant-based (PB) counterparts (n = 62) available in the UK market. Back-of-pack (BoP) and front-of-pack (FoP) nutrition label information, nutrition and health claims, and nutrient profiling model scores were compared. BoP labels revealed that meat products had higher protein, fat, and saturated fat content (*p* = 0.029), whilst PB alternatives were higher in dietary fibre and carbohydrates (*p* < 0.001). Red colour coding (FoP) for fat and saturated fat (‘high’) was more prominent in meat products (23 and 35%, respectively), and the red meat category had the most products with high fat and saturated fat content. Only 15% of meat products made nutrition claims compared to 40% of PB alternatives, and none included health claims. Most red meat PB alternatives made a nutrition claim, all related to the protein content (34%). The nutrient profiling model indicated that 74% of the PB alternatives were ‘healthy’ compared to 60% of the meat products. No association was found between the product type (meat/PB) and healthiness, except for the red meat products, which showed a significant negative association (*p* = 0.005), suggesting that these products corresponded to less healthy options. Therefore, PB alternatives can be considered as healthier substitutes for meat products.

## 1. Introduction

Vegan diets are characterised by consuming plant-based (PB) foods and omitting animal-derived products [1]. In the last decade, there has been an increased surge in the the popularity of vegan lifestyles, with more than 700,000 people worldwide taking part in the international Veganuary campaign in 2023 [2]. In 2024, there was an increase in the number of self-declared vegans in the UK by 79% compared to 2023 [3].

The recent trend of adopting ‘sustainable diets’ significantly impacted the worldwide PB foods market, which, in 2024, saw an increase of 2.8 billion US dollars compared to 2023 [4]. By 2025, the global market of PB products is projected to increase to approximately USD 77.8 billion from an anticipated 44.2 billion US dollars in 2021 [5]. Despite recent technological improvements [6], manufacturers face challenges concerning the sensory attributes of PB products because meat products’ distinctive flavour and textural attributes cannot be easily replicated. As a result, manufacturers rely on further processing methods and additive use, leading to lengthy on-pack ingredient lists that consumers consider unhealthy [6,7,8].

The nutrition information displayed on food labels informs consumers about the healthiness of the food product, influencing purchase [9]. Under UK law, pre-packed foods must provide nutritional information on the packaging, and the inclusion of a back-of-pack (BoP) label is compulsory. The Foods Standards Agency (FSA) advises retailers across the UK to provide front-of-pack (FoP) traffic light (TL) nutrition information on a range of products. The FoP TL system rates fat, saturated fat, sugar, and salt (per serving) content as ‘low’, ‘medium’, or ‘high’ by colour-coding these as ‘green’, ‘amber’, or ‘red’, respectively [10].

Nutrient profile models (NPMs) are used to evaluate the nutritional quality of food products. They serve as scientific foundations for legislative, labelling, tax, and educational purposes, and the food industry uses them as standards for product reformulations [11,12]. The FSA developed the UK version of the NPM in 2005, aiming to help the Office of Communications regulate food advertising to children. The model uses a simple point-based system, crediting points based on the quantities of nutrients, fruit and vegetables available in 100 g of a food or drink. This allows the products to be classified as ‘healthy’ or ‘less healthy’. The reliability of different NPMs as nutrition research tools has been widely accepted and supported by a growing body of literature; therefore, NPMs have been used in many nutrition research methodologies [13,14,15].

This paper aims to assess the nutritional quality of meat products and their PB alternatives available on the British market by (1) comparing BoP and FoP nutritional information, (2) determining the healthiness of the products using nutrient profiling, and (3) reviewing nutrition and health claims declared on the packaging. To our knowledge, a few UK studies have compared the nutritional quality of meat and PB products [16,17,18,19].

## 2. Methods

### 2.1. Data Collection

This cross-sectional study reviewed the information on the packaging of processed meat products (red meat, chicken and fish on their own or as part of a meal, e.g., chicken drumsticks, bacon, tuna, mincemeat, burgers, sausages, pasties, pies, ready meals, etc. (n = 62)) and their PB alternatives (n = 62) available in the UK market. Data was collected during December 2022 via random sampling using internet keyword searches (‘beef’, ‘chicken’, ‘fish’, ‘pork’, ‘duck’) from the websites of supermarkets that trade in the UK (Asda, Iceland, M&S, Ocado, Sainsbury’s, Tesco and Waitrose). The meat products were selected, and corresponding PB alternatives were then searched for using suitable keywords (‘vegan meat alternatives’, ‘vegan fish alternatives’, ‘no chicken’, ‘meat free’, ‘vegan bacon’, ‘vegan pie’, ‘vegan rolls’, ‘fishless’, ‘porkless’, ‘vegan tuna’, ‘vegan sausages’). The products were manufactured by different companies, and some carried the supermarket’s own labels. The manufacturer of the meat products and their corresponding PB alternatives were very rarely matched (detailed information about the products are provided in Appendix A). The manufacturers of the branded products were Asda (Asda Limited, Sutton, England), Birds Eye (London, UK), Charlie Bigham’s (London, UK), Dopsu (ABP Food Group, Liverpool, UK), Good Catch (Gathered Foods, Russellville, OH, USA), Gressingham (Gressingham Foods, Suffolk, UK), Heck (Heck Food Ltd., Yorkshire, UK), Itsu (Itsu Grocery Ltd., London, UK), Linda McCartney’s (Hain Celestial Group, Fakenham, UK), Magpye (Magpye Ltd., Newcastle Upon Tyne, UK), Morrisons (Wm Morrison Supermarkets PLC, Bradford, UK), M&S (Marks and Spencer Group P.L.C., London, UK), Naughty Vegan (Naughty Vegan Limited, Leamington Spa, UK), On the Go (Sainsbury’s Supermarkets, London, UK), OUMPH (Livekindly UK Ltd., Bicester, UK) Plant Chef (Tesco PLC, London, UK), Plant Living (Waitrose Ltd., Bracknell, UK), Plant Menu (ALDI UK, Birmingham, UK), Plant Pioneers (Sainsbury’s Supermarkets, London, UK), Pukka (Pukka Pies Ltd., Leicestershire, UK), Quorn (Monde Nissin Corporation, North Yorkshire, UK), Richmond (Pilgrim’s Food Masters Ltd., Hyde, UK), Sainsbury’s (Sainsbury’s Supermarkets London, UK), Tesco (Tesco PLC, London, UK), Taste and Glory (Pilgrim’s Food Masters Ltd., Hyde, UK), The Jolly Hog (The Jolly Hog Group Ltd., Bristol, UK), The Tofoo Co. (The Tofoo Company Ltd., North Yorkshire, UK), THIS™ (The Aircraft Factory, London, UK), Vegan Zeastar (Vegan Finest Foods, Rijswijk, The Netherlands), Vivera (Vivera UK Ltd., Warwick, UK), Waitrose ( (Waitrose Ltd., Bracknell, UK) and Wicked Kitchen (Wicked Foods Ltd., Corby, UK) (presented in alphabetical order).

The nutrition information presented on the BoP (energy and nutrient content) and voluntary FoP labels (traffic light colour coding indicating low, medium, and high levels of nutrients), the ingredients, portion size and weight of the products, and any nutritional and health claims declared on the packaging were collected.

### 2.2. Analysis of Nutritional Labels and Claims

The products’ mean energy and nutrient (fat, saturated fat, carbohydrate, sugar, protein, fibre and salt) content per 100 g (n = 124) were recorded and compared. The meat products were also divided into three sub-categories based on the primary protein source: red meat, poultry and fish to facilitate more specific comparisons (since they differ in nutritional content). BoP and FoP data were compared for each category (meat vs. PB) and sub-categories against their PB alternatives (i.e., red meat versus red meat alternatives, poultry versus poultry alternatives etc.).

For products that did not have FoP labels, colour coding for fat, saturated fat, sugar and salt per portion size was determined using the Department of Health guidance [20]. This information was used to compare the colour coding assigned to the products, which were assessed individually (i.e., meat versus plant alternative) and also within sub-categories.

All products were also screened for any nutritional claims (i.e., nutrition and health claims), and comparisons were made within sub-categories.

### 2.3. Nutrient Profiling

The nutrient profiling was performed using the Nutrient Profile Model Online Calculator designed by the University of Leeds [21]. The NPM classifies the foods into ‘healthy’ or ‘less healthy’ according to their nutritional composition and beneficial food components such as fruits, vegetables, or nuts (FVNs). This classification relies on points awarded for each nutrient/food component, as indicated by the relevant tables. Energy, saturated fat, total sugar, and sodium are classified as ‘A’ nutrients, while FVN content, fibre, and protein are ‘C’ nutrients. The overall NPM score is calculated by subtracting the total A points from the total C points. Foods with scores less than 4 are considered ‘healthy’ [22].

*Total ‘A’ points = (points for energy) + (points for saturated fat) + (points for sugars) + (points for sodium)*

*Total ‘C’ points = (points for % fruit, vegetable & nut content) + (points for fibre [either NSP or AOAC]) + (points for protein)*

where quantitative ingredient declarations (QUIDs) were unavailable on the packaging, the percentage of FVN ingredients was approximated using their position in the ingredient list as per the guidance in Table 1. This technique has been previously used by Poon et al. [23] in a study assessing different NPMs.

### 2.4. Statistical Analysis

Data were analysed using IBM SPSS Statistics Software, version 27. Descriptive statistics (mean and SD) were used to summarise nutritional data per 100 g. Significant differences were determined at the *p* < 0.05 level.

Independent sample t-tests were used to compare meat products’ mean energy and nutrient content with their PB counterparts. Comparisons were also made between product sub-categories (i.e., red meat, poultry and fish) and their PB counterparts. The chi-square test was used to examine the association between the type of product (meat products/PB alternatives) and healthiness.

## 3. Results

### 3.1. Nutritional Assessment

Mean values for energy and nutrients are presented in Table 2. The energy content of the meat products was slightly higher for meat products (202.0 ± 9.4 kcal/100 g) than their PB alternatives (194.0 ± 9.0 kcal/100 g); however, this difference was not significant (*p* = 0.377).

Red meat products (*n* = 31) had significantly higher energy content (+37 kcal/100 g, *p* < 0.001) than their PB counterparts. Interestingly, the energy provided by the PB poultry alternatives was higher than that of poultry products (+33 kcal/100 g, *p* = 0.026). The energy contents of fish products (*n* = 9) and their PB alternatives were not significantly different (*p* = 0.097).

The comparison of nutrient values (per 100 g) revealed higher values of fat, saturated fat, and protein in meat products (*p* = 0.029; *p* < 0.001 and *p* < 0.001, respectively). In contrast, PB alternatives were higher in carbohydrates, sugars, fibre, and salt, but only the fibre content was significantly different (*p* < 0.001). The salt content was comparable between the two groups (1.1 g/100 g). Poultry products were higher in saturated fat, sugar and protein, with only protein values differing significantly (*p* < 0.001). However, fat, carbohydrate, fibre and salt were significantly higher in their PB alternatives (*p* = 0.010; *p* = 0.022; *p* < 0.001; *p* = 0.030, respectively). Fish products had significantly higher protein content (*p* = 0.009), whilst their PB alternatives were significantly higher in carbohydrates and salt (*p* < 0.001 and *p* = 0.012, respectively).

### 3.2. Front of Pack Nutrition Labels

**Figure 1:**
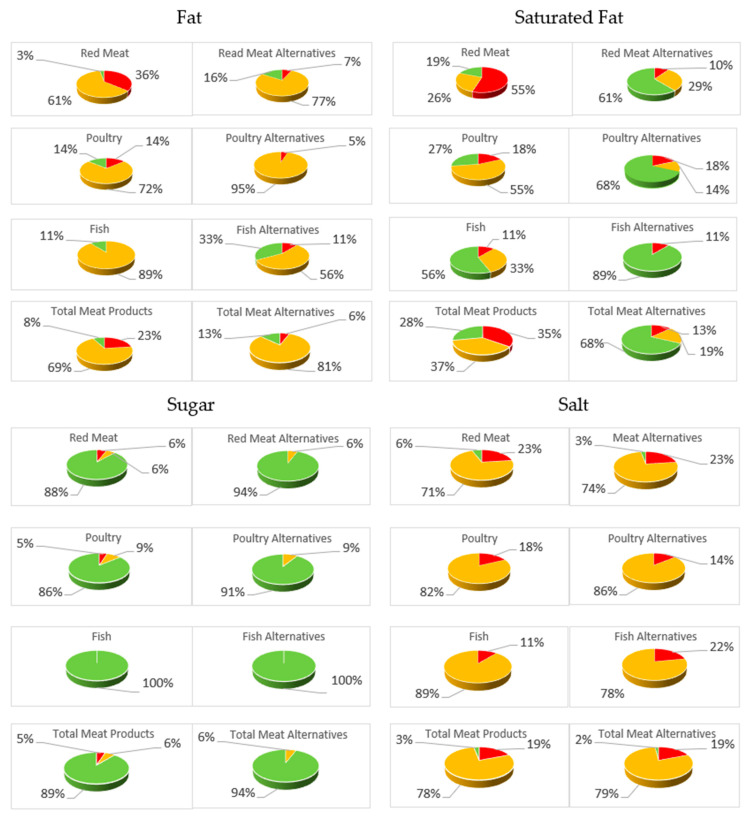
Summary of FoP TL information for meat and PB products. (Red: High, Amber: Medium and Green: Low).

A summary of the FoP TL information is shown in Figure 1. The results showed that 23% of the meat products were high in fat compared to 6% of the PB alternatives. More than one-third (35%) of the meat products had high levels of saturated fat, which was higher than that of the PB alternatives (13%). The meat group also had fewer products with a low saturated fat content (28%) than the PB alternatives (68%).

High sugar content was observed in only 5% of the meat products and none of the PB products. Interestingly, 19% of meat and PB products contained high salt levels.

**Figure 2:**
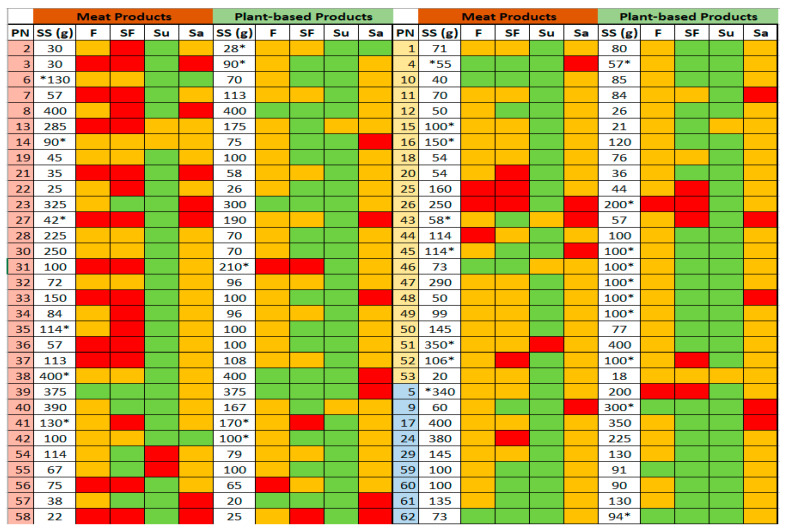
FoP TL colour coding for individual products per serving (red: high, amber: medium and green: low). * No FoP information was available; values were calculated using nutrient composition per 100 g and serving size information. PN: Product number (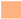: red meat, 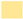: poultry, 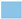: fish), SS: Serving size, F: Fat, SF: Saturated fat, Su: Sugar, Sa: Salt.

The FoP TL information for individual product against its PB counterpart within each sub-category is presented in Figure 2, along with the serving size for each product (please see Appendix A for further details). FoP TL information was unavailable for 14 of the meat products and 15 of the PB alternatives; therefore, it was calculated using the 2016 UK Food Standards Agency’s guide on creating FoP nutrition labels for pre-packed foods sold via retail outlets [20].

### 3.3. Nutrition and Health Claims

The PB meat alternatives had more nutrition claims (40%) than the meat products (15%) (Table 3). Over a third (34%) of the PB alternatives included a protein-related claim, with most claims made for red meat alternatives. Red meat and poultry products carried fat and protein-related claims, whilst omega-3 fatty acids-related claims were made for fish products. A ‘low energy’ claim (‘24 kcal per slice’) was observed in only one poultry product, and two meat products had multiple nutrition claims (‘low fat/high protein’; ‘low fat/high protein/low energy’).

Fibre-related claims were recorded for 24% of the PB products. Saturated-fat-related claims were made for 11% PB products but for none of the meat products. One fish PB product made an omega-3 related claim and three PB products claimed, ‘Source of vitamin B12’ and ‘Source of iron’.

None of the products included a health claim.

### 3.4. Healthiness of Foods

**Figure 3:**
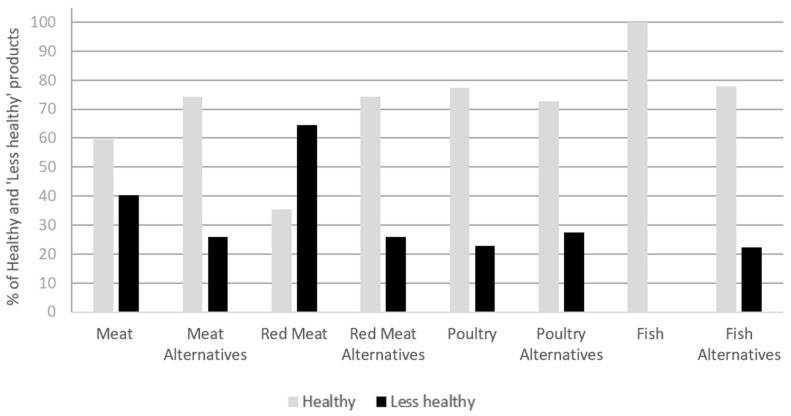
Categorisation of products as ‘Healthy’ and ‘Less healthy’ by nutrient profiling (*p* values for meat, red meat, poultry and fish categories are *p* < 0.086, *p* < 0.005, *p* < 0.728 and *p* < 0.471, respectively).

The results of the NPM analysis and the associations between product type (meat/PB) and healthiness can be found in Figure 3. More than half of the meat products (60%, 37 products) and 74% of the PB alternatives (46 products) were found to be ‘healthy’, but the association between product type and ‘healthiness’ was not statistically significant. Within the different meat sub-groups (red meat, poultry and fish), the only significant association between product type and ‘healthiness’ was observed for red meat products (*p* < 0.005), suggesting that only the red meat alternatives are healthier than their meat counterparts.

## 4. Discussion

### 4.1. Nutritional Value

The energy content of the meat and PB products did not differ significantly. This was consistent with similar UK [18,24] and Spanish [25] studies that evaluated the nutritional value of meat products and their PB alternatives that also reported negligible differences in energy content. On the contrary, other UK studies found that PB products had significantly lower energy content [16,17,19]. Moreover, a US-based study investigating the nutritional properties of beef burgers and their PB alternatives concluded that the PB products provided lower energy values due to significantly lower amounts of fat, saturated fat and protein [26]. The current study observed a similar trend with red meat products and PB alternatives, with the values of energy, fat, saturated fat and protein being significantly lower in the PB products.

Fats represent essential nutrients for a healthy, balanced diet, but the types of fats and amounts consumed directly impact health [27,28,29]. Total fat was higher in the meat samples, except for the fish products, where the difference was not statistically significant. Although fish products have moderate levels of total fat, it is known that fish is a good source of omega-3 fatty acids, a type of polyunsaturated fat that promotes health when consumed in adequate amounts [30]. Additionally, some fish samples included ready meals (e.g., fish pie, tuna pasta bake) and processed fish (e.g., battered fish, fish cake) that contained butter and/or vegetable oils, increasing the saturated fat content. Saturated fat, commonly found in red meat, poultry and dairy products, is unhealthy when consumed in amounts exceeding the recommended intakes, as it increases LDL cholesterol, leading to plaque build-up in the arteries and increasing the risks of cardiovascular diseases [31,32]. Red meat is known to contain higher amounts of saturated fat when compared to poultry and fish products [33,34,35]. This may explain why saturated fat content was significantly higher within the red meat group when compared to the PB alternatives. The results are in accordance with previous studies conducted in the UK [16,17,18,19,24], US [26], Sweden [36], Portugal [37] and Spain [25] that found significantly higher amounts of saturated fat in the red meat product samples.

Conversely, whilst PB foods are marketed as healthier options than traditional animal-based meats, previous research has suggested that PB products can sometimes be higher in saturated fat than their meat counterparts. The reason for the high saturated fat content in PB meat alternatives is usually associated with the addition of fats such as coconut, palm and palm kernel oil that provide technological functionalities (e.g., satiety, flavour, crispness and increased shelf life). Refined carbohydrates such as polysaccharides and starch can be used by the food industry to replace saturated fat; however, these can negatively impact the sensorial and textural properties of the foods and, therefore, consumer acceptance [38,39]. In the present study, only two PB foods contained palm oil as the main fat source, and both products (products 25 and 52) displayed red TL for saturated fat. Four of the PB products contained coconut oil as a source of fat (products 4, 16, 26 and 32). Still, only one (product no. 26) used coconut oil as the main fat source, and this was the only product containing coconut oil that displayed red TL for saturated fat. The other three products used vegetable oils (sunflower and rapeseed) as main fat sources. Out of 62 PB products analysed, only 9 were high in saturated fat, with the majority (42 products) displaying green TL for this nutrient.

Proteins are another critical nutrient for the human body, playing vital roles in tissue building and repair, immune function, enzymatic reactions and hormone production, and acting as transporters of different bodily substances [40]. Animal protein is considered a high-quality protein as it contains all the essential amino acids in sufficient amounts [41]. Although PB foods contain protein, various legumes, grains and nuts must be combined within the same product to achieve adequate intakes of all essential amino acids, as these cannot be provided by single PB protein sources (apart from soybeans) [40]. The primary protein sources in this study’s PB products were soybeans, wheat, peas, mushrooms, and mycoprotein (or a combination of these). The finding that all meat products had significantly higher protein content than the PB alternatives followed previous studies [16,17,18,19,24,25,26,36,37].

The protein quality of PB products is rarely considered by manufacturers. This is because producing an alternative product that mimics the texture and flavour of meat is of utmost importance due to issues around consumer acceptability and sales volumes. Several studies have reported that increased intake of PB foods could lead to nutritional deficiency due to the incomplete amino acid profile of these products [42,43,44]. There is a need to enhance the knowledge of proteins in PB foods by investigating their protein quality, absorption and bioavailability of the amino acids in the human body and their subsequent effect on health.

The higher values of carbohydrates reported in the PB alternatives resulted from higher fibre content within this category, as sugar levels were not statistically different. The results were similar to previous studies [16,17,18,19,24,25,26,36,37], except for fish products, which contained slightly higher (but non-significant) amounts of fibre compared to the PB alternatives. This could be explained by the ingredients present in composite fish products (such as the tuna pasta bake, cod and parsley fish cakes, XL fillets in salt and vinegar batter, breaded fish, cod fishcake with parsley sauce and fish fingers), which contained significant amounts of wheat flour, with some of these products also containing potato. In the UK, the mean daily fibre intake in the adult population is approximately 19.2 g, which is below the recommended value of 30 g [45]; therefore, including PB alternatives in diets may help improve fibre intake. A study conducted in Denmark looked at the nutritional content of PB protein products and reported that all soy (except tofu) and mycoprotein-based products contained enough fibre to make a ‘high in fibre’ claim and all pea and wheat (except seitan) based products had sufficient amounts of fibre to meet the claim ‘source of fibre’ [46]. Out of the 62 PB products analysed in the current study, 21% of the products contained sufficient amount of fibre to make a ‘high fibre’ claim (over 6 g of fibre/100 g), whilst 42% of the products had sufficient amounts of fibre to claim ‘source of fibre’ (over 3 g of fibre/100 g). Twenty-seven percent (mainly ready meals and pies) did not qualify for any fibre-related claims due to insufficient amounts of fibre, with the main ingredients within these products being potato, rice or tofu.

Sugar content was not significantly different within the two categories. This finding disagreed with another study [16], which found higher sugar levels across several meat alternative categories in the UK (budget sausages, burgers, mince and meatballs, n = 99). Per 100 g, PB products contained approximately 0.5 g more sugar than meat products, whilst the current study only reported 0.1 g more sugar in PB alternatives. Studies conducted in the US and Germany reported values up to twice as high for the sugar content of PB products compared to meat [26,47]. Sugar can be added in high amounts to PB products to improve taste and/or texture, but this practice negatively impacts the healthiness of these products [48].

Salt levels were similar between the meat products and PB alternatives. This finding follows Romao et al. [49] but disagrees with Alessandrini et al. [17], Zhang et al. [18] and Cole et al. [26], who reported significantly higher salt values in PB alternatives. Processed foods are known to be higher in salt (sodium chloride, NaCl) since it has multiple technological functions (preservative, stabiliser, flavour enhancer, or to improve texture) [50]. In PB products, hydrocolloids such as gellan gum, xanthan and methoxylated pectin create a fibrous structure and require appropriate amounts of salt to function optimally [51,52].

In Western society, high salt intakes represent a significant risk factor for cardiovascular diseases, with governments worldwide making substantial efforts to decrease salt intakes within different populations [53]. In 2020, Public Health England published the ‘Salt reduction targets for 2024’ as part of the salt reduction campaign targeted at the British population initiated in 2004. The document sets salt targets to be achieved by 2024 for 84 specific food groups that contribute the most to the salt intakes of the UK diets and include ‘meat alternatives’ as one of the groups. The salt targets for the meat alternatives category are set as follows: 0.63 g/100 g for plain meat alternatives, 0.85 g/100 g for meat-free products and 1.78 g/100 g for meat-free bacon [54]. The current results showed a mean of 1.1 g of salt per 100 g for the PB products, and only one PB alternative was below the proposed target. So, it can be argued that the products analysed did not meet the 2024 targets. Despite its preservative function, salt was still very high in PB alternatives, and this generally limited the healthiness of these products. Considering that the data in this study were collected in December 2022, further studies are needed to investigate if the 2024 targets are being achieved with the PB alternatives currently available on the market.

### 4.2. Nutrient Profiling Model (NPM)

Nutrient profiling revealed that many PB alternatives were classed as ‘healthy’, but the red meat group was the only category showing a significant association between product type and ‘healthiness’. This finding suggests that choosing PB alternatives over red meat products can represent a healthier choice and is in line with previous studies [16,17,37,55] that included larger sample sizes and more meat categories. The nutritional assessment supported these findings, as the red meat category was the only group with significantly higher levels of fat and saturated fat compared to the PB alternatives and with the highest percentage of red colour coding compared to the poultry and fish products. Moreover, the significantly higher fibre content of the red meat alternatives contributed to the ‘healthiness’ of these products.

Although there was no statistically significant difference between product type and ‘healthiness’ of the poultry and fish categories, the PB alternatives within these categories were generally less healthy than the meat counterparts due to their slightly higher salt content.

PB products can be improved to have a better nutritional profile. Andreani et al. [6] highlighted the importance of further explorations of PB protein blends to increase the bioavailability of amino acids. PB proteins have lower anabolic capacities than animal proteins; however, combining different PB proteins could improve the composition of essential amino acids and help achieve the body’s needs [56]. Several PB products in this study combined different PB protein sources, the most common being soya with wheat protein and pea protein with wheat protein. Additionally, Berrazaga et al. [57] suggested that fortifying cereal products with legumes can improve the amino acid composition of these foods, promoting better protein retention in the body.

The current findings showed that most of the meat and PB products had moderate to high salt levels, suggesting that further work can be carried out to decrease the salt content of these products, making them healthier. Although salt reduction may negatively influence consumer acceptance and product characteristics, several strategies can be implemented to reduce this impact. Examples can be using salt replacers, product reformulations and the modification of the size and structure of salt molecules (e.g., spray drying, electromagnetic atomisation drying, ultrasound, etc.) [58]. Only one meat product in this study (Morrisons Reduced Fat Pork Sausages 400 g) used potassium chloride (KCl) as a salt replacer, but this was used in addition to salt (NaCl), and the product’s salt content was categorised as amber (medium).

### 4.3. Health and Nutrition Claims

Health and nutrition claims help consumers make informed food choices and promote healthy eating. They also serve as a marketing tool for food manufacturers [59]. Neither the meat products nor the PB alternatives in this study carried any health claims. Nearly all PB products (22 out of 25) made a claim that was protein-related since high protein content might be a critical factor influencing consumers’ decision to purchase PB alternatives. Alcorta et al. [60] supported this idea, emphasising that some consumers could be sceptical about adopting vegan diets as such foods might not have the same nutritional properties as animal products, contributing less towards healthy and sustainable diets.

Many of the nutritional claims on PB products were related to their fibre (high) and/or saturated fat (low) content. Some PB alternatives made claims related to omega-3 fatty acids, vitamin B12, and iron, making them promising alternatives to meat products by providing comparable quantities of these nutrients as their meat counterparts. A Swedish study [36] that assessed PB meat alternatives (n = 142) reported that 73% of the products had a health claim, and a higher percentage of products met the requirements to claim ‘source of protein’ (97%), ‘source of fibre’ (83%) and ‘low in saturated fat’ (71%).

As the number of self-declared vegans is rapidly growing in the UK, from 1.4 million in 2023 to 2.5 million in 2024 [3], UK manufacturers should consider adding nutrition claims to more products that meet the requirements. Offering consumers additional information can contribute to healthier and sustainable diets and, at the same time, promote the products. The manufacturers can explore the possible claims PB meat alternatives can carry. It is also worth investigating if the products without a claim do not qualify or if the manufacturer deliberately omits the claims from the label.

The present study is the first to simultaneously assess the nutritional content, nutritional profiles, and nutritional claims of three categories of meat products (red meat, chicken, and fish) and their PB alternatives available on the British market. In addition, it provides a comprehensive analysis of the three product categories in terms of nutritional content and claims. However, the limitations of the study must also be noted. Firstly, the study included 62 products and their PB alternatives and this may not accurately represent all the products available in the UK market and the variability among different brands and product types. Secondly, the products were classified as ‘red meat’, ‘chicken’ and ‘fish’ products, but a further breakdown of these groups (e.g., sausages, mince, burgers, ready meals, and cold cuts) might have provided a better understanding of the healthiness and nutritional variability of different types of meat products and their PB alternatives. In addition, comparison based on sub-categories did not allow the inclusion of minimally processed PB foods such as tofu, tempeh and seitan, which could be a valid and healthy alternative to meat-based products. Finally, not all supermarkets present in the UK market were included. Further research is necessary to assess these foods’ protein quality and micronutrient content and how nutritional quality is influenced by the selection of ingredients and processing techniques used in producing PB meat alternatives.

## 5. Conclusions

This study assessed the healthiness of meat products and their PB alternatives using several nutritional tools. Nutritional assessment revealed that meat products were less healthy when compared to PB alternatives due to their higher fat and saturated fat content. On the contrary, PB products were ‘healthier’ because of their high fibre content, a nutrient associated with positive health effects. The significantly lower protein content within the PB category suggests that food manufacturers and retailers should improve existing formulations by carefully considering both protein content and quality. Data collected suggests that PB vegan products can be proposed as healthier alternatives to meat products, especially red meat products. This study contributes to the existing literature in this field and can be used by the food industry to increase the healthfulness of meat and PB products. The study also provides a nutritional assessment of PB foods versus meat-based foods, which may help UK consumers make healthier choices.

## Figures and Tables

**Table 1:** Criteria for estimating FVN% content of foods, as per Poon et al. [23]. FVN: Fruit, vegetable and nut.

FVN Content (%)	‘In-House’ Criteria Based on the Ingredients List Used to Estimate FVN Content
**Non-concentrated FVN ingredients**	
≤40	FVN is not one of the first two ingredients.
>40	FVN is the second ingredient.
>60	FVN is the first ingredient, but non-FVN ingredients contribute substantially to the product’s weight.
>80	FVN is the first ingredient, and only FVN ingredients contribute substantially to the product’s weight.
**Concentrated FVN ingredients**	
≤40	FVN is not one of the first three ingredients.
>40	FVN is the second ingredient, but the amounts of the first and second ingredients are not similar.
>60	FVN is the first ingredient, but non-FVN ingredients appear substantially to the product’s weight.
>80	FVN is the first ingredient, and only FVN ingredients contribute substantially to the product’s weight.

**Table 2:** Energy and nutrient values (per 100 g) of meat products and their PB counterparts.

	Meat Products	Plant-Based (PB)	*p*-Value
**Meat (n = 62) vs. PB alternative**			
Energy (kcal)	202.0 ± 9.4	194.0 ± 9	0.377
Fat (g)	10.9 ± 1	8.9 ± 0.6	0.029
Saturated fat (g)	3.8 ± 0.4	1.9 ± 0.3	<0.001
Carbohydrates (g)	9.5 ± 1.1	14.4 ± 1.2	<0.001
Sugar (g)	1.8 ± 0.3	1.9 ± 0.2	0.742
Protein (g)	16.3 ± 1.6	12 ± 0.8	<0.001
Fibre (g)	1.2 ± 0.2	4.1 ± 0.3	<0.001
Salt (g)	1.0 ± 0.1	1.1 ± 0.1	0.151
**Red meat (n = 31) vs. PB alternative**			
Energy (kcal)	221.0 ± 14.7	184.0 ± 10.5	<0.001
Fat (g)	13.7 ± 1.6	8.3 ± 0.8	<0.001
Saturated fat (g)	5.3 ± 0.6	2 ± 0.4	<0.001
Carbohydrates (g)	8.3 ± 1.5	12.3 ± 1.4	<0.001
Sugar (g)	1.5 ± 0.3	2.1 ± 0.4	0.105
Protein (g)	16 ± 1.2	12.9 ± 1.0	<0.001
Fibre (g)	1.2 ± 0.2	4.2 ± 0.5	<0.001
Salt (g)	1.1 ± 0.2	1.1 ± 0.1	0.828
**Poultry (n = 22) vs. PB alternative**			
Energy (kcal)	183.0 ± 63.0	216.0 ± 87.0	0.026
Fat (g)	8.0 ± 1.2	10.6 ± 1.2	0.010
Saturated fat (g)	2.3 ± 0.5	2.0 ± 0.5	0.503
Carbohydrates (g)	9.6 ± 1.7	14.3 ± 2.4	0.022
Sugar (g)	2.0 ± 0.5	1.8 ± 0.4	0.747
Protein (g)	21.9 ± 4.0	13.4 ± 1.5	<0.001
Fibre (g)	1.2 ± 0.3	4.4 ± 0.4	<0.001
Salt (g)	0.9 ± 0.1	1.2 ± 0.1	0.030
**Fish (n = 9) vs. PB alternative**			
Energy (kcal)	176 ± 13.2	177 ± 20	0.966
Fat (g)	8.0 ± 1.3	6.7 ± 1.5	0.359
Saturated fat (g)	1.7 ± 0.4	1.0 ± 0.5	0.101
Carbohydrates (g)	12.5 ± 2.6	22.1 ± 2.7	<0.001
Sugar (g)	1.3 ± 0.3	1.6 ± 0.6	0.656
Protein (g)	13.1 ± 2.2	5.7 ± 1.8	0.009
Fibre (g)	3.6 ± 2.6	2.5 ± 0.6	0.712
Salt (g)	0.8 ± 0.1	1.0 ± 0.1	0.012

**Table 3:** Nutrition claims for meat products and PB alternatives.

Meat Products		Plant-Based Alternatives	
Product No	Claim	Product No	Claim
18	30% Less fat	6	High protein
19	50% Less fat	18	High protein/source of fibre/75% Less fat
32	High protein	20	Source of protein/High fibre
20	Low fat/High protein/Low energy	21	High protein
43	High protein	22	High protein/source of fibre
45	Low fat/High protein	27	High protein/High fibre
51	Low fat	28	High protein/Low saturated fat
9	High omega-3	32	High protein/source of fibre
60	Source of omega-3	34	Source of protein/source of fibre
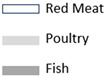		36	High protein/High fibre/Low saturated fat
		37	High protein/source of fibre
		46	High protein/Source of vitamin B12/Source of iron
		47	High protein/Source of vitamin B12/Source of iron
		54	High protein
		4	High protein
		10	High protein/High fibre/Source of vitamin B12/Source of iron
		12	High protein/Low saturated fat/source of fibre
		14	High protein/Low saturated fat
		43	High protein/High fibre/Low fat
		44	Source of protein/High fibre
		50	Source of protein/Low saturated fat
		59	Source of fibre
		60	Source of fibre/Low saturated fat
		61	Source of fibre/Low saturated fat
		62	High protein/High omega-3

## Data Availability

The original contributions presented in the study are included in the article, further inquiries can be directed to the corresponding author.

## Appendix A

**Table A1:** Product Information.

	Meat Products	Plant-Based Alternatives
1	Tesco 72 Breaded Chicken Nuggets 1 kg	Tesco Breaded Meat-Free Nuggets 320 g
2	Tesco 50 Sausage Rolls 750 g	Linda McCartney’s Vegetarian Mini Sausage Rolls 168 g
3	Tesco Sliced Chorizo Sausage 150 g	Linda McCartney’s Vegetarian Chorizo & Red Pepper 270 g
4	Heck Italian Chipolatas 340 g	Tesco Meat Free Vegan Chipolatas 300 g
5	Charlie Bigham’s Fish Pie 340 g	M&S Mushroom Pie 200 g
6	Asda Tender Beef Stir-Fry Strips 350 g	Dopsu No Beef Pieces 280 g
7	Birds Eye Beef Burgers with Onions 227 g	Tesco 2 Meat Free Burgers 226 g
8	Asda Beef Stew & Dumplings 400 g	Waitrose No Beef Bourguignon with Mash 400 g
9	Asda Flavoursome Oak Smoked Salmon 120 g	Vegan Zeastar Sashimi Zalmon 300 g
10	Asda Sliced Chargrill Style Chicken Breast 120 g	This Isn’t Chicken Plant-Based Pieces 170 g
11	Sainsbury’s British Turkey Meatballs 400 g	Tesco 12 Meat Free Balls 336 g
12	Cajun British Chicken Breast Slices 160 g	Quorn Roast Chicken Style Slices 105 g
13	Sainsbury’s Pulled Pork with BBQ Sauce 569 g	Tesco BBQ Pulled No-Pork 350 g
14	Gressingham Crispy Aromatic Half Duck 550 g	Linda McCartney’s Vegetarian Shredded Hoisin Duck 300 g
15	Itsu Korean BBQ Beef Gyoza 240 g	Asda 10 Vegetable Gyoza 210 g
16	Morrisons Chicken & Bacon Pasta Bake 400 g	Tesco (Wicked Kitchen) Nana’s Lasagne 700 g
16	Young’s 2 XL Fillets in Salt & Vinegar Batter 300 g	Birds Eye Fishless Battered Fillets x2 240 g
18	Morrisons The Best Reduced Fat Sausages 400 g	THIS Isn’t Pork Plant Based Sausages 270 g
19	Morrisons Reduced Fat Pork Sausages 400 g	Tesco 6 Herby Bangers 300 g
20	Oakpark Smoked Turkey Breast Rashers 150 g	Asda 10 Meat-Free Bacon Style Rashers 180 g
21	Waitrose Classic Frankfurters 350 g	Plant Menu 4 Meat Free Hot Dogs 240 g
22	Waitrose Corned Beef 4 Slices 100 g	Quorn Vegan Roast Beef Style Slices 105 g
23	Waitrose No.1 Beef Bourguignon 650 g	Tesco (Wicked Kitchen) Beautiful Bourguignon 300 g
24	Waitrose Italian Recipe Tuna Pasta Bake 380 g	Sainsbury’s Roasted Vegetable Pasta Salad 225 g
25	M&S 2 Chicken Kyivs 320 g	Tesco 10 Meat Free Bangers in Duvet 220 g
26	M&S Chicken, Leek & Smoked Bacon Pie 500 g	Magpye Chick’n, Leek & Bacun Pie 200 g
27	The Jolly Hog Pigs in Blankets 210 g	This Isn’t Pork Plant Based Pigs in Blankets 190 g
28	M&S British Basted Beef Joint Boneless 450 g	Dopsu No Beef Pieces 280 g
29	Aldi Melt in The Middle Cod Fishcake 290 g	Waitrose No Fishcakes 260 g
30	Aldi Meal Kit Teriyaki Beef Stir Fry 500 g	The Tofoo Co. Teriyaki Tofu 280 g
31	Crestwood Quiche Lorraine 400 g	Pukka Vegan Minced Steak & Onion Pie 210 g
32	Sainsbury’s 12 Beef Meat Balls 350 g	Taste and Glory 12 Meat Free Vegan Balls 288 g
33	M&S 12 British Meat Balls 300 g	Linda McCartney’s Vegetarian Meat Balls 240 g
34	Tesco Finest 12 British Meat Balls 336 g	Taste & Glory 12 No Meat Balls 288 g
35	Ocado 12 Beef Meat Balls Italian Style 336 g	OUMPH Balls 280 g
36	Birds Eye 4 Original Beef Burgers 227 g	Quorn Vegan Hot & Spicy Burgers 246 g
37	M&S Organic 2 British Beef Burgers 225 g	Quorn Ultimate Meat Free Burger 227 g
38	Iceland Family Beef Lasagne 1.6 kg	Plant Pioneers Vegan Lasagne 400 g
39	Bisto Spaghetti Bolognese 375 g	Bistro Vegan Spaghetti Bolognese 375 g
40	M&S Roast Beef 390 g	Sainsbury’s Three Nut Roast 500 g
41	Iceland 2 Beef Pasties 260 g	Naughty Vegan 2 No Beef Pasties 360 g
42	Sainsbury’s Lamb Mince 500 g	Linda McCartney’s Vegemince 500 g
43	Heck Simply Chicken Chipolatas 340 g	Heck meat Free Chicken Chipolatas 300 g
44	Birds Eye 2 Chicken Burgers 228 g	Birds Eye Chicken free Burgers 200 g
45	Heck Simply Chicken Burgers 228 g	Linda McCartney’s 2 Quarter Pounder Burgers 227 g
46	Sainsbury’s Teriyaki Chicken Breast Kebabs 290 g	Vivera Veggie Greek Kebab 175 g
47	Asda Slow Cooked Chicken Shawarma 580 g	Vivera Plant Shawarma Kebab 175 g
48	M&S BBQ Chicken Kebabs 100 g	OUMPH Kebab Spiced 280 g
49	Tesco Ready to Roast Chicken Drumsticks 460 g	Wicked Kitchen No Chicken Drumettes 250 g
50	M&S Crispy Chicken Nuggets 290 g	Quorn 15 No Chicken Nuggets 300 g
51	Lovelife Keralan Chicken Biryani 350 g	Sainsbury’s Vegetable Biryani with Basmati Rice 400 g
52	Pukka Chicken & Mushroom Pie 212 g	Pukka Vegan Chicken and Mushroom Pie 210 g
53	Sainsbury’s Duck Spring Rolls 200 g	Sainsbury’s 10 No Duck Spring Rolls 180 g
54	Sainsbury’s 8 Pork Sausages 454 g	Richmond 8 Meat Free Sausages 336 g
55	M&S 6 Free Range Pork Sausages 400 g	Linda McCartney’s Vegetarian Sausages 270 g
56	Tesco 2 Melton Mowbray Pork Pies 150 g	Tesco 2 Vegan Porkless Pies 130 g
57	M&S Cured Smoked Ham 115 g	Quorn Vegetarian Ham Slices 100 g
58	Tesco Pepperoni Slices 160 g	Quorn Vegan Pepperoni Slices 100 g
59	Birds Eye 2 Breaded Fish Fillets 200 g	Quorn 2 Breaded Fishless Fillets 200 g
60	Birds Eye 8 Fish Fingers 200 g	Quorn Fish Fingers 200 g
61	Asda 2 Cod & parsley Fish Cakes 270 g	Morrisons Thai Style Fishless Cakes 260 g
62	Tuna Chunks in Spring Water 145 g	Good Catch Plant Based Tuna Naked in Water 94 g
